# Functional Neurological Disorder and the Risk of Social Detachment

**DOI:** 10.1192/j.eurpsy.2023.1439

**Published:** 2023-07-19

**Authors:** M. Gheis, K. Lenk

**Affiliations:** 1 Psychiatry, University of British Columbia; 2 Adminstration, Program for Functional Neurological Disorder, Victoria, Canada

## Abstract

**Introduction:**

Functional Neurological Disorder (FND) is associated with altered social-emotional cognition. Social isolation is a recognized complication of FND and is a perpetuating factor for this condition. Limited data is available on the severity and determinants of social isolation and detachment in FND.

**Objectives:**

To assess the prevalence, severity and determinants of social detachment in patients with FND.

**Methods:**

This is a study of 32 consecutive referrals to a specialist FND service for adults.

We analyzed the study subjects’ scores on the Social Detachment PAI trait subscale. High social detachment scores on this subscale are recognized to occur in socially isolated people and those with difficulty interpreting the normal emotional nuances of interpersonal behaviour.

We evaluated the correlation between scores on the Social Detachment subscale and the symptomatological pattern of FND. Subsequently, patients were classified into two groups: those who subjectively evaluated their symptoms as visible (primarily those with Functional motor FND and Non-epileptic Attack Disorder) and those who subjectively evaluate their symptoms as not significantly visible (predominantly sensory FND).

We evaluated the correlation between subjective sense of symptom visibility, demographic and comorbidity variables on one hand and social detachment on the other hand.

We examined the correlation between the social detachment scores and difficulties interpreting emotional expressions as detected on the Perception of Emotions Test (POET).

**Results:**

In a normative standardization population sample the 90th percentile *T* score of the PAI Social Detachment Subscale was 54. In the study sample of patients with FND the mean score was high, exceeding the 90th percentile at 59 ( *p*< 0.05).

In terms of comorbidity, we identified a high-risk ratio of social detachment in patients with FND who also have a concurrent diagnosis of Somatization Disorder (Risk ratio = 4.1; 95% CI, 1.6 to 10).

There was no statistically significant correlation between patients’ demographic variables and Social Detachment score, nor was there a statistically significant correlation between the phenomenology and the visibility of Functional Neurological Disorder (motor, sensory, cogniform, non-epileptic attack disorder or mixed) and social detachment.

We found no correlation between subjects’ scores on the Perception of Emotions Test and their scores on Social Detachment.

**Conclusions:**

Social detachment is a significant feature of FND, particularly in those with a concurrent diagnosis of somatization disorder. Rehabilitation focused on restoring social function may be an essential intervention. Social detachment in this population may not be related to understanding nuances of emotional expression, nor is it related to the visibility of FND symptoms. Further research is needed to understand social cognitive processes in FND, specially when associated with somatization disorder.

**Disclosure of Interest:**

None Declared

